# *Wdr74* Is Required for Blastocyst Formation in the Mouse

**DOI:** 10.1371/journal.pone.0022516

**Published:** 2011-07-25

**Authors:** Marc Maserati, Melanie Walentuk, Xiangpeng Dai, Olivia Holston, Danielle Adams, Jesse Mager

**Affiliations:** Department of Veterinary and Animal Science, University of Massachusetts, Amherst, Massachusetts, United States of America; University of Illinois at Chicago, United States of America

## Abstract

Preimplantation is a dynamic developmental period during which a combination of maternal and zygotic factors program the early embryo resulting in lineage specification and implantation. A reverse genetic RNAi screen in mouse embryos identified the WD Repeat Domain 74 gene (*Wdr74*) as being required for these critical first steps of mammalian development. Knockdown of *Wdr74* results in embryos that develop normally until the morula stage but fail to form blastocysts or properly specify the inner cell mass and trophectoderm. In *Wdr74*-deficient embryos, we find activated Trp53-dependent apoptosis as well as a global reduction of RNA polymerase I, II and III transcripts. In *Wdr74*-deficient embryos blocking Trp53 function rescues blastocyst formation and lineage differentiation. These results indicate that Wdr74 is required for RNA transcription, processing and/or stability during preimplantation development and is an essential gene in the mouse.

## Introduction

Preimplantation development in the mouse is a time of dynamic change in which the fertilized egg becomes a pluripotent embryo that subsequently develops into a blastocyst with two distinct cell lineages. This developmental period is characterized by three major transitions, each of which entails pronounced changes in the pattern of gene expression. The first transition is the maternal-to-zygotic transition (MZT) that serves three functions: (1) to destroy oocyte-specific transcripts, (2) to replace maternal transcripts that are common to the oocyte and early embryo with zygotic transcripts and (3) to facilitate the reprogramming of the early embryo by generating novel transcripts that are not expressed in the oocyte [1]. Zygotic gene activation initiates during the late 1-cell stage at some genes and throughout the genome by the 2-cell stage [1], [2]. Coincident with genome activation is the acquisition of a chromatin-based transcriptionally-repressive state [3], [4] and more efficient use of TATA-less promoters [5], which are likely to play a major role in establishing the appropriate patterns of gene expression required for proper development.

Up to the 8-cell stage, individual blastomeres are loosely associated. During compaction, adhesive interactions form between blastomeres to generate a tightly organized mass of cells [6]. Accompanying this morphological transition are pronounced biochemical changes through which blastomeres acquire characteristics resembling somatic cells, reflected in such features as ion transport, metabolism, cellular architecture, and gene expression patterns. The appearance of gap and tight junctions at the late 8-cell stage results in an epithelium that is essential for proper development [6], [7].

Following compaction, cleavage divisions allocate cells to the inside of the developing morula. These inner cells of the morula give rise to the inner cell mass (ICM) from which the embryo proper is derived. The outer cells differentiate exclusively into the trophectoderm (TE), which generates extraembryonic tissues. The TE is a fluid transporting epithelium that is responsible for forming the blastocoel cavity, which is essential for continued development and differentiation of the ICM [8], [9]. Overt cellular differentiation first occurs in the blastocyst and is characterized by differences in gene expression between the ICM and TE cells. For example, expression of Pou5f1 (Oct4) and Fgf4 [10], [11], [12] become restricted to the ICM, while expression of *Bex1*, the imprinted *H19* gene and *Cdx2*, an ortholog of the Drosophila homeotic Caudal (*Cad*) gene, are restricted to the TE in the preimplantation embryo [13], [14], [15]. Proper specification of these distinct lineages is required for blastocyst formation and facilitates hatching from the zona pellucida and implantation into the uterine epithelium [reviewed in [16]].

In order to expand our understanding of the genes required for these critical developmental events, we initiated an RNAi based screen during mouse preimplantation. Based on the simple assumption that a dynamic change in a given gene's expression level may be indicative of temporal specific function, we analyzed published micro-array data sets [17], [18], [19] for genes with dynamic expression patterns during preimplantation stages. We first used an unbiased *in silico* approach to merge published data sets in order to select genes based on preimplantation expression pattern alone, irrespective of associated gene ontology [20]. Genes with known roles during preimplantation and genes known to be required for cell viability were then removed as candidates. We next tested the preimplantation requirement of each of the remaining candidate genes by microinjection of gene-specific long double-stranded RNAs (dsRNAs) into fertilized 1-cell zygotes and subsequent culture *in vitro* to the blastocyst stage, an approach which has been used successfully during preimplantation and does not elicit an interferon response or significant off-target effects [21]. We maximized our screening potential through injection of pools of up to 5 different dsRNAs in order to knockdown 5 separate genes simultaneously. Pooled dsRNAs that resulted in a phenotype were then injected singly to determine the gene responsible. Here we present data showing that the WD Repeat Domain 74 gene (*Wdr74*) is required during preimplantation development.

*Wdr74* is characterized by six WD40 repeats, which are minimally conserved structural motifs of approximately 40 amino acids that often terminate in a tryptophan-aspartic acid (WD) dipeptide [22]. To date, no studies have functionally characterized *Wdr74*. Analysis of the predicted Wdr74 protein using functional domain finding algorithms reveals no identifiable functional domains, besides the WD repeats, providing little insight. *Wdr74* is a single copy gene that is very well conserved across mammalian species with all vertebrates having one orthologous locus of at least 77% amino acid identity [23], [24]. Interestingly, there are no homologous loci in mammals with significant amino acid similarities besides the WD repeat domains, indicating the unique nature of Wdr74. Although very little is known about *Wdr74*, the superfamily of WD40 repeat proteins have been implicated in a wide variety of cellular functions, such as cell division, cell fate determination, gene transcription, transmembrane signaling, mRNA modification, and vesicle fusion [25]. The function of WD-repeats has been shown to mediate protein-protein interactions during complex assembly [26] possibly explaining the wide array of functions that are ascribed to genes containing them. Additionally, the importance of WD-repeat proteins is evident: the sequence has been conserved across all species in eukaryotes [Reviewed in [26]], and WD repeat motifs have also been identified in prokaryotes as well [27], [28]. Here, we present data describing the developmental and molecular consequences of loss of *Wdr74* function.

## Materials and Methods

### Ethics Statement

All animal studies were approved by the Animal Care and Use Committee, University of Massachusetts, protocol 2010-0021.

### Embryo Production and collection

B6D2F1 females (Jackson Laboratories, Stock# 100006) received an intra-peritoneal injection of pregnant mare serum gonadotropin (PMSG; 5 IU/animal), followed by stimulation with human chorionic gonadotropin (hcG; 5 IU/animal) 48 hours later. Mice were mated with B6D2F1 males. Embryos (0.5 dpc) were collected via flushing the infundibulum with M2 medium.

### Microinjection

Injection and holding micropipettes were pulled from borosilicate capillary tubes with a Sutter p-87 glass puller (Sutter Instruments). The injection needle tip was cut to make a 1–2 µM diameter opening and the needle was then bent to 15° using a microforge (DeFronbrun). Microinjection was performed using a piezo impact-drive injector (PrimeTech) mounted on a Diaphot inverted microscope (Nikon) with Hoffman Modulation Contrast objectives and condenser. Micromanipulation of embryos was performed using TransferMan NK 2 (Eppendorf,) in M2 medium (Chemicon) with 0.01% PVP (Sigma). Each embryo was injected with approximately 5 pL dsRNA. After all embryos were injected, they were transferred into microdrops of M2 medium for 10 minutes at 37°C before deposition in KSOM medium for extended culture.

### Embryo Culture

Embryos were cultured in 35 mm petri dishes (Falcon) in 30–50 µL drops of KSOM (Chemicon) under 3 mL mineral oil (Fisher Scientific) in 5% CO_2_, 5%O_2_ and 90% N_2_ at 37°C in a humidified incubator.

### dsRNA *in vitro* transcription

For T7-RNA polymerase mediated double stranded RNA (dsRNA) production, *Wdr74*, *Trp53* and Green Fluorescent Protein (GFP) specific PCR primers were designed using 40 base-pair oligos that contained the T7 binding sequences followed by gene specific sequences as follows: GfpF 5′- TAATACGACTCACTATAGGGCACATGAAGCAGCACGACTT -3′ and GfpR 5′-TAATACGACTCACTATAGGGTGCTCAGGTAGTGGTTGTCG-3′, Wdr74F 5′ - TAATACGACTCACTATAGGGCGGAATGATTGGCTTGATCT -3′ and Wdr74R 5′ - TAATACGACTCACTATAGGGAGGGTACTTGGTTGGGCTCT -3′ ; Trp53F 5′ TTATACGACTCACTATAGGGCACGTACTCTCCTCCCCTCA-3′ and Trp53R 5′-TAATACGACTCACTATAGGGTACCTTATGAGCCACCCGAG -3′. Oligos were purchased from IDT. dsRNAs were created using the MEGAscript T7 *in vitro* transcription kit (Ambion) followed by DNase treatment (Roche) and purification using NucAway Spin Columns (Ambion) and Phenol/Chloroform extraction. dsRNAs were re-suspended in Nuclease-Free water (Integrated DNA Technologies) and diluted to 400 ng/µL for microinjection. dsRNA was stored at −80°C until use.

### Pyronin Y RNA staining

Embryos were fixed in 4%PFA for 10 minutes, rinsed twice in PBS with 0.3% PVP (PBS/PVP) and stained in PBS/PVP Hoechst (IDENT Hamilton Thorne) 40 µg/mL for 10 minutes. Embryos were then directly transferred to PBS/PVP 5 µM Pyronin Y (ACROS) for 10 minutes. The embryos were then rinsed three times in 400 µL PBS plus 0.3% PVP before being mounted in VectaShield (Vector Labs) on glass slides and evaluated for epifluorescence with a TE2000S inverted microscope (Nikon). All photographs were taken using a Retiga EXi camera (Photometrics) using National Instruments Elements (National Instruments) image capture software under phase contrast and epifluorescence.

### RNA Quantification

The Agilent RNA 6000 Pico kit was used according to manufacturer's recommendations. RNA was extracted from embryos using the High Pure RNA Isolation Kit (Roche) and resuspended to 1.6 embryo equivalents per microliter (EE/µL). Samples were run in triplicate. RNA concentration was also assessed by NanoDrop spectrophotometer according to manufacturers' instructions.

### RNA isolation and cDNA synthesis

Total RNA was extracted from single and pooled embryos using the High Pure RNA Isolation Kit (Roche) according to manufacturer's recommendations. First strand cDNA was prepared from total RNA using the qScript cDNA Synthesis Kit (Quanta) according to the manufacturers' instructions.

### Quantitative Real-Time PCR

Real-time RT-PCR was performed using 0.75 embryonic equivalents with Taqman probe based gene expression assays from Applied Biosystems as follows: 2X Quanta PerfeCTa Supermix Low ROX, 20X Vic-labeled *ActB* (#4352341E) or *GapD* (#4352339E), 20X Gene Expression Assay (*Bax* Mm00432050_m1; *miR-125* TM002198; *miR-721*- #TM001657; *Oct4* Mm00658129_gH; *Tead4* Mm01189836_m1; *Trp53* Mm01731287; *Wdr36* Mm00620161_m1; *Wdr74* Mm00506573_m1; *Rnu6* TM1973; *Sno110* TM1230.

*Snord65* TM1228; *Mvp* Mm00453676_m1) and water to total 20 µL. Reactions were run in triplicate on a Stratagene Mx3005p Real-Time PCR machine with a thermal profile of: 1 cycle of 50°C for 30 seconds, 1 cycle of 95°C for 2 minutes, then 40 cycles of 15 seconds at 95°C and 30 seconds at 60°C.

### Reverse-Transcriptase PCR

PCR was performed using 0.75 embryonic equivalents, 2X RubyTaq mastermix, 10 mM of both forward and reverse primers, and water to total 20 µL. The *Wdr74* forward primer used was 5′-CGGAATGATTGGCTTGATCT-3′ and the reverse primer was 5′-AGGGTACTTGGTTGGGCTCT-3′. PCR reactions were performed in a Biorad MyCycler machine for 35 cycles of 95°C for 30 seconds, 60°C for 30 seconds, 72°C for 30 seconds with a final extension of 2 minutes at 72°C.

### Immunofluorescence

Embryos were fixed in 4% paraformaldehyde for 30 minutes at room temperature, washed three times in phosphate buffered saline (PBS) containing 0.1% Tween-20, and permeabilized with PBS containing 1% TritonX-100 for 30 minutes at room temperature. Embryos were then blocked for 1 hour at room temperature in 2% bovine serum albumin (BSA) in PBS. Primary antibodies were added in appropriate dilutions [1∶500 rabbit anti WDR74 (Sigma SAB1102237); 1∶50 rabbit anti-Trp53 polyclonal (Cell Signaling Technologies), 1∶200 rabbit anti-Ecadherin polyclonal (Abcam), 1∶200 rabbit anti-Oct4 polyclonal (Abcam), 1∶200 mouse anti-Cdx2 monoclonal (BioGenex), 1∶200 rabbit anti-H3K4me3 polyclonal (Abcam), 1∶200 Rabbit Anti-Fibrillarin (Abcam, ab5821), 1∶100 Rabbit Anti-CTD PolII (Abcam, ab5095)] in PBS/BSA (2%) for 1 hour at room temperature. After the embryos were washed three times with PBS containing 0.1% Tween-20, secondary antibodies (Alexa Fluor 546 Anti Rabbit – Molecular Probes A-11035; Alexa Fluor 488 Anti Rabbit – Molecular Probes A-21206; Alexa Fluor 488 Anti Mouse – Molecular Probes A-11001) were added at a 1∶200 dilution in PBS/BSA and incubated for 1 hour at room temperature. 4′,6-Diamidino-2-phenylindole (DAPI) was used as a marker to stain nuclear DNA at a concentration of 1∶10,000 for 10 minutes, then the embryos were washed three times with PBS containing 0.1% Tween-20, and mounted in water under VWR micro cover glass. As negative controls, embryos were treated similarly, without primary antibodies. Embryos were imaged using a Retiga EXi camera (Photometrics) using National Instruments Elements (National Instruments) image capture software under phase contrast and epifluorescence.

### Blastomere Counting

Double-blind cell counting was repeated by two individuals using DAPI-stained nuclei and the NIS Elements BR software.

## Results

### *Wdr74* is required for blastocyst formation

**Figure 1:**
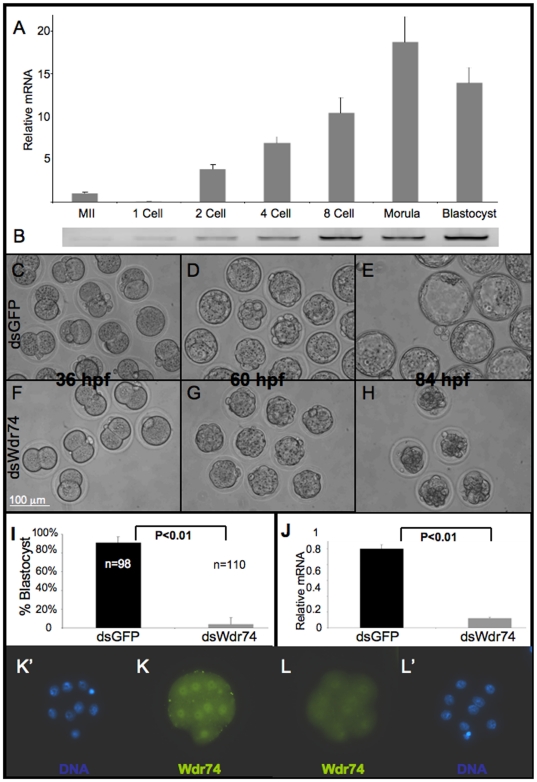
Wdr74 is required for blastocyst formation. **A.** Quantitative RT-PCR analysis of endogenous *Wdr74* mRNA during preimplantation development. **B.** RT-PCR with *Wdr74* intron-spanning primers confirms relative abundance of transcripts observed by qRT-PCR. **C–H.** Microinjected and cultured embryos photographed at 36, 60, and 84 hours post fertilization. Control dsGFP-injected embryos show normal development and form blastocysts by 84 hpf (C–E). dsWdr74 injected embryos develop normally to the morula stage (F–G) but fail to make blastocysts (H). **I.** Quantification of percent 2 cell embryos that develop to the blastocyst stage by 84 hpf (# blastocysts/# 2-cell ×100). **J.** qRT-PCR of Wdr74 transcripts indicates robust RNAi mediated knockdown due to microinjection of dsWdr74. **K.** Immunofluorescence of Wdr74 in morula stage dsGFP embryos shows nuclear localization; which is drastically reduced in dsWdr74 embryos of the same stage (L). hpf, hours post fertilization. Results of student T-test shown, error bars represent standard deviation. All data shown normalized to embryo equivalents; MII, Metaphase II oocyte. Scale bar in F representative for C–H. K′ and L′ show DAPI signal (DNA) from the same embryos shown in K and L, respectively.

In order to determine the temporal expression of *Wdr74* during preimplantation development, we used both quantitative reverse-transcriptase PCR (qRT-PCR, Figure 1A) and intron spanning qualitative RT-PCR (Figure 1B) to visualize endogenous mRNA levels at various preimplantation stages. As shown in Figure 1, *Wdr74* is expressed at relatively low levels in MII oocytes and 1-cell embryos and increases through subsequent cleavage stage divisions. The peak of mRNA expression occurs at the morula stage, with a slight decrease in blastocyst embryos (Figure 1A), suggesting a key function during the morula to blastocyst transition. Additionally, immunofluorescence with an antibody directed against human WDR74 showed no specific signal until the 8-cell stage in wild type embryos (not shown), when weak nuclear localization is observed. In morula and blastocysts, Wdr74 is evident within nuclei of all cells (Figure 1K). This pattern of protein expression perfectly correlates with the endogenous mRNA levels, and suggests no Wdr74 function until late 8-cell/early morula stages.

In order to remove *Wdr74* activity, we injected dsWdr74 RNA into the cytoplasm of 1-cell embryos. In all experiments presented, control embryos were injected with dsGFP in order to similarly stimulate the RNAi machinery and show specific effects of loss of *Wdr74*. *Wdr74* knockdown embryos (hereafter referred to as dsWdr74 embryos) were able to compact and develop normally to the morula stage with no obvious differences in morphology or rate of development compared to controls (compare Figure 1C and D with F and G). By 84 hours post fertilization in culture (hpf), 91% (89/98) of control embryos have initiated blastocoel formation (Figure 1E and I). However, only 4% (4/110) of the dsWdr74 embryos showed any evidence of a blastocoel cavity (Figure 1H, I). The majority of dsWdr74 embryos showed visible signs of degeneration by 84 hours post fertilization, with morphologically evident dying and irregular cells (Figure 1H).

**Figure 2:**
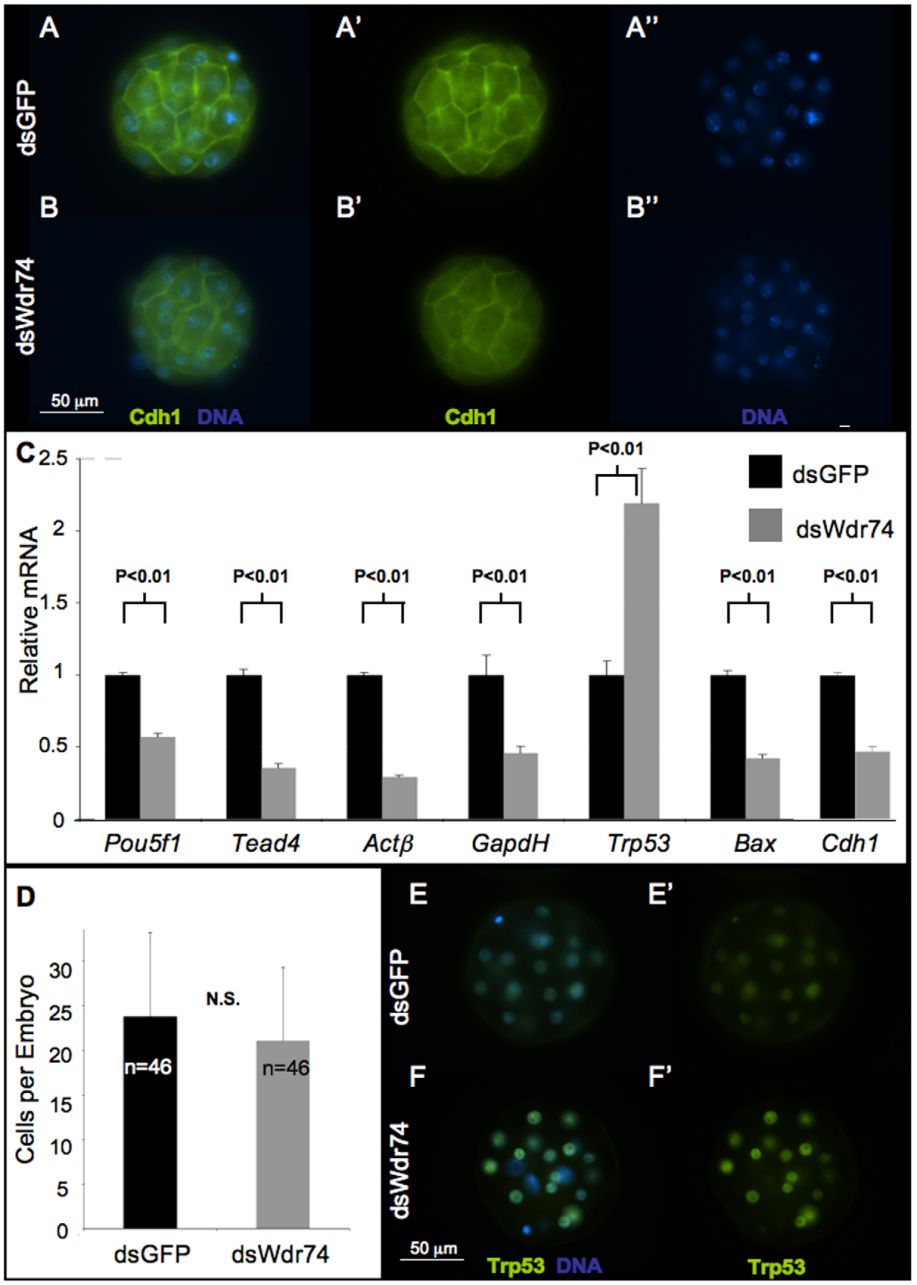
Gene expression in dsWdr74 morula. **A–B.** E-cadherin (Cdh1) localization by immunofluorescence marks blastomere cell-cell adhesion as expected in dsGFP morula (A). E-Cadherin is appropriately localized but present at reduced in dsWdr74 morula (B). **C.** qRT-PCR assays show reduced RNA polymerase II derived transcripts of *Pouf51*, *Tead4*, *Actβ*, *GapdH*, *Bax*, and *Cdh1* but *Trp53* shows an increase in transcripts in Wdr74-deficinet embryos. **D.** The average number of cells in dsGFP and dsWdr74 morula is not significantly different. **E–F. **Localization of Trp53 by immunofluorescence shows a marked increase of Trp53 protein in dsWdr74 embryos (compare F to E), consistent with the increase in *Trp53* mRNA. Results of student T-test shown, error bars represent standard deviation. All data shown normalized to embryo equivalents. N.S., not significant. Scale bar in B and F representative for A–B and E–F, respectively.

We confirmed robust knockdown of dsWdr74 by qRT-PCR with cDNA from pooled control and experimental embryos at the morula stage (Figure 1J). Furthermore, immunofluorescence showed drastic reduction in dsWdr74 morula stage embryos indicating functional knockdown of Wdr74 (compare Figure 1K and L), and consistent reduction of both mRNA and protein using this approach. As stated above, dsWdr74 embryos appear morphologically normal through the morula stage. However, immunofluorescence revealed that the blastomeres of dsWdr74 morula have reduced E-cadherin protein at cell-cell contacts relative to controls (compare Figure 2A–C and D–F). Nonetheless, the appropriate localization of E-cadherin suggests that knockdown morula blastomeres are contacting each other appropriately in preparation for compaction and cavitation. Consistent with a peak in *Wdr74* activity during the morula to blastocyst transition (Figure 1), these results indicate that a molecular and cellular phenotype is present in dsWdr74 morula, 1 day before the embryos degenerate.

### Lineage specific expression and apoptosis in dsWdr74 morula

In order to explore possible reasons for the blastocyst failure and embryo death, we examined markers of lineage specification and apoptosis. As shown in Figure 2C, we find an overall decrease in both *Oct4* and *Tead4* transcripts, known markers of ICM and TE, respectively (results shown relative to embryo equivalence). Importantly, we also observe a reduction in the “housekeeping” transcripts, *β-actin* and *Gapdh*, within dsWdr74 embryos (Figure 2C). The reduced transcript levels could be the result of either reduced mRNA or reduced cell numbers per embryo. Counting of cells in dsGFP and dsWdr74 morula revealed no reduction in cell number in dsWdr74 morula (Figure 2D), suggesting either global reduction of transcription or a loss of mRNA due to defects in stability and/or processing of nascent transcripts.

Consistent with the blastomere cell death observed at 84 hours, we find an increase in transcripts of the apoptotic marker *Trp53* (p53) in dsWdr74 morula (Figure 2C). However, we did not observe a similar increase in *Bax* levels, a gene thought to be downstream of *Trp53* [29]. Instead, *Bax* transcripts show a similar decrease in abundance as *Oct4*, *Tead4*, *Gapdh* and *β-actin*. Supporting the qRT-PCR results, we observe increased Trp53 protein within the cells of dsWdr74 morula (compare Figure 2E and F).

### Co-injection of dsWdr74 and dsTrp53 permits blastocyst formation

**Figure 3:**
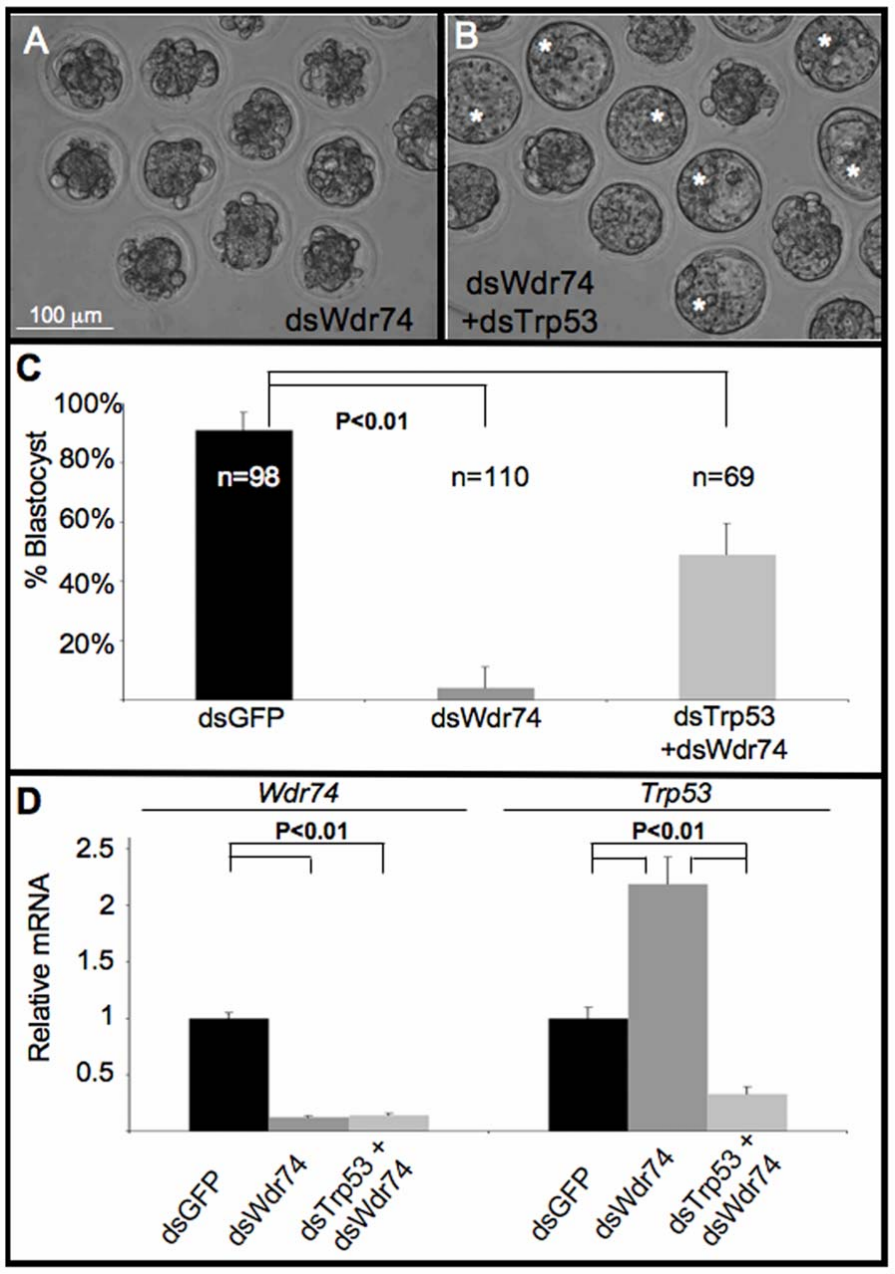
Blocking *Trp53* permits blastocyst formation in Wdr74-deficient embryos. **A–B.** Morphological evaluation of dsWdr74-injected and dsWdr74+dsTrp53 co-injected embryos at 84 hpf. dsWdr74 embryos do not develop past the morula stage (A). Reduction of *Trp53* permits differentiation of Wdr74-deficient blastocysts (B). **C.** Percent of 2-cell embryos reaching the blastocyst stage by 84 hpf in dsGFP, dsWdr74 and dsWdr74+dsTrp53 co-injected embryos. **D.** qRT-PCR confirms knockdown of *Wdr74* and *Trp53* as expected. Results of student T-test shown, error bars represent standard deviation. All data shown normalized to embryo equivalents.

**Figure 4:**
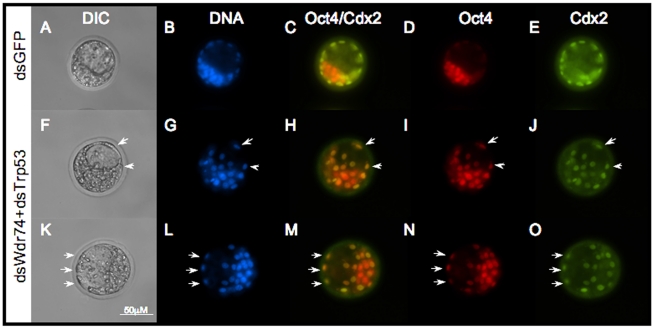
Lineage specification in dsWdr74/dsTrp53 blastocysts. **A–H.** Immunofluorescence localizes Oct4 and Cdx2 to the inner cell mass and trophectoderm, respectively, in dsGFP embryos (A–E). In dsWdr74/dsTrp53 rescued blastocysts (2 shown, F–J and K–O), Cdx2 is expressed and Oct4 is reduced (but present) in some TE-like cells (arrows in F–O). Scale bar in K representative for all panels. DIC, differential interference contrast microscopy.

Because *Wdr74*-deficient embryos showed increased levels of *Trp53*, we attempted to block apoptosis by knocking-down *Trp53* by co-injection of dsWdr74 and dsTrp53. Reduction of *Trp53* in dsWdr74 embryos rescued blastocyst formation in 49% of embryos (Figure 3A–C). For this experiment we added an additional control of injecting dsTrp53 alone, which had no adverse consequences on development to the blastocyst stage (data not shown). All injection groups (dsGFP, dsWdr74, dsTrp53 and dsWdr74/dsTrp53) developed normally to the morula stage. qRT-PCR confirmed expected knockdown in each group (Figure 3D). As expected, dsWdr74 embryos failed to form blastocysts and remained at the morula stage with obvious morphological signs of dying cells. However, 49% (34/69) of the dsWdr74/dsTrp53 co-injected embryos were able to successfully form blastocoel cavities with morphologically distinct ICM and TE cells during the culture period (asterisks, Figure 3B). This rescue of blastocyst formation (from 4% to 49%) suggests that dsWdr74 knockdown embryos are capable of differentiating ICM and TE cells, but fail to do so due to Trp53-induced apoptosis. However, immunofluorescent staining shows that the majority of TE cells in these “rescued” blastocysts express *both* Oct4 and Cdx2 (arrows, Figure 4) indicating that although morphological differentiation has occurred, molecular patterning has not been properly established. Furthermore, many cells within the ICM of dsWdr74/dsTrp53 blastocysts inappropriately express Cdx2. Consistent with reduced transcript levels in the absence of *Wdr74*, the levels of both Oct4 and Cdx2 protein were reduced in dsWdr74/dsTrp53 embryos. Many of these double knockdown “rescued” blastocysts contain morphologically abnormal and/or dying cells, indicating that while blastocyst formation does occur, viability was not rescued. It is also worth noting that Wdr74/Trp53-deficient blastocysts do not expand as wild type embryos do, and remain in “early” blastocysts stages, reminiscent of the Cdx2 null embryo phenotype, which form early blasts but fail to maintain epithelial integrity of the TE [30].

### Global reduction of RNA in Wdr74 deficient embryos

**Figure 5:**
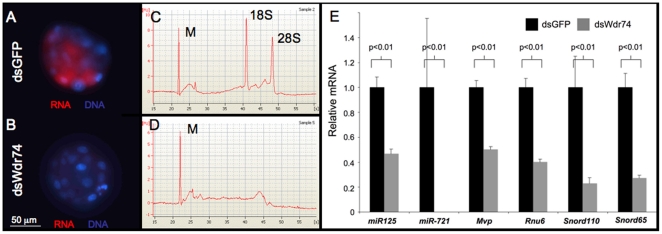
Global loss of RNA in dsWdr74 embryos. **A–B.** Hoechst 33322 (blue) and Pyronin Y (red) stains bind to DNA and RNA, respectively, in dsGFP (A) and dsWdr74 (B) embryos. Note the dramatic decrease in Pyronin Y fluorescence in the absence of Wdr74. **C–D.** Bioanalyzer electropherograms from total RNA extracted from dsGFP (C) and dsWdr74 embryos (D) show that 18s and 28s ribosomal RNAs are nearly absent in Wdr74-deficient embryos. **E.** Relative levels of microRNAs *miR-125* and *miR-721* and RNA polymerase III derived transcripts, *Mvp*, *Rnu6*, *Snord110* and *Snord65* in dsGFP and dsWdr74 morula. Results of student T-test shown, error bars represent standard deviation. All data shown normalized to embryo equivalents.

Based on the observation of reduced transcripts of all genes examined (except *Trp53*) when *Wdr74* is knocked-down (Figure 2), we assessed global RNA levels in morula stage embryos using Pyronin Y, a fluorescent molecule that binds RNA [31]. We find a drastic decrease in Pyronin Y fluorescence in morphologically normal dsWdr74 morula at 60 hpf, indicating a significant reduction of RNA in Wdr74-deficient embryos (compare Figure 5A and B). It is worth noting that approximately 8 hours earlier (∼52 hpf), no qualitative differences were detectable in Pyronin Y fluorescence, suggesting a rapid decrease in RNA at the time when dsWdr74 embryos begin to compact. To confirm and quantify these results, RNA was extracted from equal numbers of dsGFP and dsWdr74 embryos (50 each) and assessed using a Bioanalyzer. As shown in Figure 5C, 18S and 28S peaks are clearly observed in RNA extracted from dsGFP-injected embryos at the morula stage. However, these peaks are entirely absent in RNA extracted from Wdr74-deficient morula (Figure 5D).

As with the qPCR assays, we used equal numbers of embryo equivalents in order to compare dsGFP and dsWdr74 samples. For the Bioanalyzer results shown, precisely 1.7 embryo equivalents of total RNA was used for both dsGFP and dsWdr74 samples, in triplicate (Figure 5C and D). Consistent with reduced but not absent gene specific transcripts (Figure 2C), many minor peaks are present in the electropherogram from the dsWdr74 extracted RNA, indicating that some RNA is present. Given these results, we also assessed RNA samples with a Nanodrop spectrophotometer, which indicated extraction of ∼5.8 ng total RNA from each dsGFP morula, while only ∼0.66 ng was recovered from each dsWdr74 embryo. This reduction in total RNA is consistent with the qPCR results that show between 2 and 5-fold reduction of gene specific transcripts.

Since we observed reduction of transcripts generated by both RNA polymerase I (ribosomal RNAs) and RNA polymerase II (coding transcripts in Figure 2), we also assessed levels diverse types of RNA species. We examined two microRNAs (*mir-125* and *miR-721*) expressed during preimplantation [32] and four RNA polymerase III dependent transcripts, major vault protein (*Mvp*), U6 small nuclear RNA (*Rnu6*), and two small nucleolar RNAs [*Snord110* and *Snord65* [33], [34]]. Consistent with a global RNA reduction, we observe significant decrease of all of these gene products in dsWdr74 embryos (Figure 5E).

## Discussion

Here we have shown the molecular and developmental consequences of loss of Wdr74 function *in vivo*. Our results indicate that Wdr74 is required during preimplantation development and suggest that this poorly studied protein plays an essential role in global RNA transcription, processing and/or stability across the genome. We observe a similar reduction of transcripts produced by all RNA polymerases (I, II and III) suggesting that Wdr74 may be a common protein component of all three polymerase complexes. Interestingly, a human WD containing protein, Wdr92, has been identified in as a common component of RNA polymerase complexes (reviewed in [35]). Furthermore, we observe reduction of both intron-containing and single-exon transcripts, indicating that the global reduction is not due to a failure of pre-mRNA splicing which has also been shown required for blastocyst formation [36], [37]. Similarly, RNA polymerase I deficient embryos develop to morula but fail to form blastocysts [38], supporting the possibility that Wdr74 is an essential component of RNA polymerase complexes.

The increase in *Trp53* mRNA and protein indicates that the basic cellular processes of transcription, mRNA processing and translation can (and does) occur in the absence of Wdr74. It does remain possible that Wdr74 is absolutely required for transcription or translation but that there is sufficient Wdr74 protein present at the time of mRNA knockdown to allow for transcription and/or translation of Trp53, a possibility consistent with the reduction, but not absence of Wdr74 protein in knockdown embryos. However, we do observe Cdx2 protein, albeit at reduced levels in dsWdr74/dsTrp53 embryos. Cdx2 protein is not consistently present at high levels in blastomeres until late morula/early blastocyst stage [39]. This is precisely when we observe strongest reduction in *Wdr74* transcripts in dsWdr74 embryos. Given that we do observe Cdx2 protein in Wdr74 deficient cells of “rescued” blastocysts (Figure 4), we conclude that Wdr74 function is not absolutely required for transcription or translation. Similarly, we observe reduced but present E-cadherin protein, which is required during preimplantation for blastomere compaction and formation of functional TE [40]. The reduction in E-cadherin may contribute the failure to form blastocysts, as it is also required for a properly polarized TE [41]. However, because dsWdr74 blastomeres appropriately localize E-cadherin and knockdown embryos do compact normally, we believe that the reduction in E-cadherin is a consequence – not a cause of the phenotype in Wdr74-deficient embryos.

Another possible explanation for the reduction of RNA could be defects in nucleolus function/structure. We do not observe morphological defects in nucleolus formation or size based on visual morphology or Fibrillarin localization (not shown), nor do we observe overt cellular defects prior to day 3 in culture, the time at which dsWdr74 embryos have failed to form blastocysts. The fact that we observe a reduction in all transcripts and protein during a stage when dsWdr74 embryos appear morphologically normal suggests that Wdr74 is essential during preimplantation for RNA transcription, processing or stability and that as proteins degrade below functional thresholds, Wdr74-deficient blastomeres undergo Trp53 dependent apoptosis. The rapid reduction of RNA coincident with Trp53 activation occurs at the precise developmental stage when we observe the highest *Wdr74* transcript levels in wild-type embryos. As we do not observe appreciable levels of maternal transcripts or protein, our results indicate that Wdr74 is not required until the morula-to-blastocyst transition.

Two other WD-repeat family members have recently been shown to be required for early development. Gallenberger et al. demonstrated that a lack of the WD-repeat protein 36 (Wdr36) caused preimplantation embryonic lethality [42]. Investigation in tissue culture showed that depletion of Wdr36 led to apoptotic cell death, increased expression of *Cdkn1A*, *Trp53*, and *Bax* transcripts, and a substantial decrease in 21S ribosomal RNA, the precursor of the 18S rRNA. These studies concluded that Wdr36 is an essential protein for nucleolar processing and maturation of the 18S small subunit ribosomal RNA.

Knockdown of the WD repeat 82 gene (*Wdr82*) has been shown to recruit the Setd1A histone H3 lysine 4 trimethylation complex to the Ser5-phosphorylated C-terminal domain of RNA polymerase II [43], and more recent work has dissected the role of Wdr82 in the methyltransferase complex during embryonic development [44]. Although neither Wdr36 nor Wdr82 share significant protein similarity with Wdr74, their similar phenotypes underscore the requirement for WD proteins during preimplantation development.

While the precise mechanism of Wdr74 remains to be determined, our results offer the following conclusions: 1) *Wdr74* is expressed from the zygotic genome and is required for morula to blastocyst transition; 2) In the absence of *Wdr74* function there is a global reduction of RNA transcripts from RNA polymerase I, II and III; 3) Loss of *Wdr74* activates Trp53 dependent apoptosis; 4) When Trp53 function is blocked, dsWdr74 embryos are capable of morphological differentiation and blastocyst formation although molecular patterning of lineage specification remains defective.
